# Sexual and reproductive health and rights in emergencies

**DOI:** 10.2471/BLT.16.173567

**Published:** 2016-05-02

**Authors:** Ian Askew, Rajat Khosla, Ugochi Daniels, Sandra Krause, Clare Lofthouse, Lale Say, Kate Gilmore, Sarah Zeid

**Affiliations:** a Family, Women’s and Children’s Health, World Health Organization, avenue Appia 20, 1211 Geneva 27, Switzerland.; b United Nations Population Fund, New York, United States of America (USA).; c Women’s Refugee Commission, New York, USA.; d Office of the High Commissioner for Human Rights, Geneva, Switzerland.; e United Nations Foundation, New York, USA.

The World Health Organization defines health emergencies as sudden-onset events from naturally occurring or man-made hazards, or gradually deteriorating situations through which the risk to public health steadily increases over time.^1^ In recent years, conflict, violence and disasters have brought a dramatic rise in the number of displaced people, both within and across national borders. According to the Office of the United Nations High Commissioner for Refugees, in 2014, there were 60 million internally-displaced people and international migrants, half of whom come from Afghanistan, Somalia and the Syrian Arab Republic.^2^ The average time spent in displacement has now reached 20 years.^2^ Women and girls are affected disproportionately in both sudden and slow-onset emergencies^3^ and face multiple sexual and reproductive health challenges in emergency contexts. There are an estimated 26 million women and girls of reproductive age living in emergency situations, all of whom need sexual and reproductive health services.^4^

In countries designated by the Organisation for Economic Co-operation and Development as fragile states,^5^ the estimated lifetime risk of maternal mortality is 1 in 54.^6^ Three-quarters of the countries with maternal mortality ratios above 300 per 100 000 live births are fragile states.^6^ With disruption and lawlessness, sexual violence often increases during emergencies, exacerbating threats to the health and survival of women and girls.^7^

The new *Global strategy for women’s, children’s and Adolescents’ Health (2016–2030)*^8^ attributes gaps in service provision to the lack of sufficient health workers in emergency settings. The strategy therefore calls for coordinated and integrated approaches to address threats to health and well-being in emergency settings.^8^ Crises expose extant weaknesses in health systems, impacting differentially on sub-populations, especially women, children and adolescents. Low resilience in health systems and an absence of quality data on women’s, children’s and adolescents’ health in emergencies hinders design and implementation of sustainable interventions.

To achieve the vision of *Transforming our world: the 2030 agenda for sustainable development* – to leave no one behind – it is imperative to protect and improve women’s, children’s and adolescents’ health and well-being in emergencies. To reach every woman, every child and every adolescent, everywhere, the global strategy recommends a series of actions. First priority is given to provision of a minimum initial service package for reproductive health^9^ by national health systems and external partners in emergencies.^8^ Second, needs and vulnerabilities have to be assessed objectively and addressed with a package of health services that includes topics such as, nutrition, human immunodeficiency virus infections, as well as water, sanitation and hygiene. Third, sustainable service delivery depends on programmes that transition from the emergency response to health systems strengthening for the long term. In addition, the strategy recognizes the critical need to ensure the safety of health workers and their facilities in conflict settings.^8^

Such actions need multi-year flexible financing from the start of an emergency, so that low- and middle-income countries can access funds when budget support from their government is no longer available. According to a recent evaluation, between 2002 and 2013, unmet funding requirements for reproductive health in emergencies amounted to 2.689 billion United States dollars.^10^

The World Humanitarian Summit, held in Istanbul, Turkey, in May 2016, is an opportunity to include the United Nations Secretary-General’s proposed agenda for humanity within the *2030 Agenda for Sustainable Development*. By endorsing the Secretary-General’s call to action, summit participants can commit to the actions proposed by the global strategy for women, children and adolescents living in emergency settings.^11^
